# Danhong Injection Alleviates Postoperative Intra-abdominal Adhesion in a Rat Model

**DOI:** 10.1155/2019/4591384

**Published:** 2019-08-19

**Authors:** Yunhua Wu, Guangbing Wei, Junhui Yu, Zilu Chen, Zhengshui Xu, Rui Shen, Ting Liang, Lu Zheng, Kang Wang, Xuejun Sun, Xuqi Li

**Affiliations:** ^1^Department of General Surgery, The First Affiliated Hospital of Xi'an Jiaotong University, Xi'an, 710061 Shaanxi, China; ^2^Department of Radiology, The First Affiliated Hospital of Xi'an Jiaotong University, Xi'an, 710061 Shaanxi, China; ^3^Department of Biomedical Engineering, the Key Laboratory of Biomedical Information Engineering of the Ministry of Education, School of Life Science and Technology, Xi'an Jiaotong University, Xi'an, 710061 Shaanxi, China; ^4^Department of Physical Examination, The First Affiliated Hospital of Xi'an Jiaotong University, Xi'an, 710061 Shaanxi, China

## Abstract

**Background:**

Among all the common complications that occur after abdominal surgery, intestinal adhesion is perhaps the most unpleasant one. However, current methods to treat and prevent intestinal adhesion are limited; thus, exploring new methods to prevent and treat intestinal adhesion is greatly needed. In this study, we demonstrated that Danhong injection (DHI) may be used as a promising method to prevent and treat intra-abdominal adhesion in a rat model.

**Materials and Methods:**

Forty-eight rats were randomly divided into six groups. Except for the sham-operated group, all rats underwent cecal abrasion to establish an adhesion model. After the operation, the rats in the DHI-treated groups received different doses of DHI via the tail vein daily, while the other group was treated with the same volume of saline solution. Seven days after the operation, all rats were sacrificed, and the degree of adhesion was evaluated by Nair's scoring system. The extent of inflammation in the adhesion tissue was detected by HE staining and the expression of tumor necrosis factor-*α* (TNF-*α*) and transforming growth factor-*β* (TGF-*β*). The collagen deposition was assessed by Sirius red staining and *α*-SMA, MMP9, t-PA, and PAI-1 levels. Oxidative stress was indicated by the level of reactive oxygen species (ROS) in adhesion tissues and by immunohistochemical labeling of Nrf2. Furthermore, rat primary peritoneal mesothelial cells (RPMCs) were treated with H_2_O_2_ and DHI, and NF-*κ*B phosphorylation was detected to illustrate the effect of DHI on oxidative stress.

**Results:**

The intra-abdominal adhesion scores were significantly decreased in the groups treated with a high dose of DHI compared with the control groups, and the degree of inflammation, fibrosis, and oxidative stress was also significantly decreased. DHI treatment significantly reduced the levels of TNF-*α*, TGF-*β*1, and PAI and increased the expression levels of MMP9, Nrf2, and t-PA in the adhesion tissues. ROS levels and NF-*κ*B phosphorylation were significantly reduced in DHI-treated RPMCs compared with the control RPMCs.

**Conclusion:**

DHI alleviates the formation of postoperative intra-abdominal adhesions by inhibiting inflammation, collagen deposition, and oxidative stress in a rat model and may serve as a promising drug to prevent intra-abdominal adhesions.

## 1. Introduction

Intra-abdominal adhesion is one of the most common complications after abdominal surgery [1]. After this operation, injury or trauma can cause inflammation, which in turn leads to collagen deposition and the development of intra-abdominal adhesion [2]. Adhesion formation is a process that involved the repair of injured peritoneal tissue; if the damaged tissue is repaired in time, adherence will not occur or limited adhesion will occur; otherwise, adhesion will occur [3]. Even though various methods have attempted to prevent postoperative adhesion, their effects are far from satisfactory. Surgeons continue to attempt to discover effective ways to prevent and treat adhesion formation [4].

Oxidative stress is used to describe the abnormal condition in which the balance between reactive oxygen species (ROS) or reactive nitrogen species (RNS) and antioxidant systems, including enzymes and nonenzyme particles, is destroyed [5]. After abdominal surgery, both an increase in ROS/RNS production and the suppression of the antioxidant system result in the development of adhesion via oxidative stress [6]. The loss of balance between these systems will not only lead to an exacerbated inflammation reaction but can also harm peritoneal tissue repair. Thus, alleviating ROS may be a potential way to prevent postoperative adhesion.

Danhong injection (DHI), a traditional Chinese medicine, was extracted from *Salviae miltiorrhizae* Radix and *Carthami tinctorii* Flos. The main bioactive constituents in DHI include salvianic acid A, salvianic acid B, protocatechuic aldehyde, and rosmarinic acid [7]. In a previous study, DHI was demonstrated to exert many effects, such as anti-inflammatory, antioxidant, anticoagulatory, hypolipidemic, antiapoptotic, vasodilatory, and angiogenesis-promoting actions [8, 9], and DHI is widely used in the treatment of cerebral ischemia and cardiovascular diseases [10]. The postoperative adhesion develops through interactions between inflammation and collagen deposition; thus, we hypothesize that DHI may have a protective effect on postoperative abdominal adhesion. Based on this hypothesis, our study was designed and conducted to show the ability of DHI to prevent postoperative adhesion by reducing inflammation, collagen deposition, and oxidative stress.

## 2. Materials and Methods

### 2.1. Experimental Animals and Reagents

Forty-eight Sprague-Dawley (SD) rats weighing 200 g to 250 g were obtained from the Experimental Animal Center of Xi'an Jiaotong University. These rats were freely housed in a thermostatic room at approximately 22 ± 2°C. DHI was purchased from Heze Buchang Pharmaceutical Co. Ltd. (Heze, China) and diluted in 0.9% saline.

### 2.2. Experimental Preparation and Design

Abdominal hair was removed one day before the operation. Anesthesia was performed by the intraperitoneal injection of 50 mg/kg phenobarbital sodium (GuideChem, Shanghai, China). Then, the skin was sterilized with pyrrolidone iodine three times. A 2 to 3 centimeter incision was created at the middle of the abdomen. Except for the sham-operated group, an intra-abdominal adhesion model was established in all the rats by scraping the abdominal and bilateral intestinal wall as described in previous studies [11]. The rats in the sodium hyaluronate (HA) group were then treated with 2 mL of HA gel (Qingdao Haitao Biochemical Co. Ltd., Qingdao, China) before the abdomen was closed. After the skin was disinfected again, the abdominal cavity was closed by an intermittent suture in two layers. Within one week after the operation, the rats in the DHI group received an intravenous injection in the tail of 0.8 mL of different doses of DHI. The doses for the DHI1 group, DHI2 group, and DHI3 group were 1 mL/kg, 2 mL/kg, and 4 mL/kg, respectively. The rats in the other three groups were injected with 0.8 mL of saline daily.

### 2.3. Assessment of General Adhesion in the Specimens

Seven days later, the rats were anesthetized as previously described and sacrificed [11]. The abdominal cavity was opened by a U-incision. The status of intra-abdominal adhesion was evaluated by Nair et al.'s [12] scoring system (Supplemental [Supplementary-material supplementary-material-1]). Then, samples of adhesion tissues were removed and divided into two parts: one part was fixed in 4% formalin, and the other part was stored at -80°C for ROS detection. Specifically, the cecum and abdominal tissues were collected from the sites with the most severe adhesion, whereas the cercal lesions and corresponding peritoneal tissues were collected from nonadhesion tissues.

### 2.4. Pathological Assessment

Twenty-four hours after soaking in formalin, the specimen was embedded in paraffin and sliced into 4 *μ*m thick pathological sections. Four incisions were randomly selected from each pathological tissue for hematoxylin and eosin (HE) staining, and the results were observed by light microscopy. Samples were evaluated based on the scoring system used in previous studies [13]. The inflammatory response was scored as follows (Supplemental [Supplementary-material supplementary-material-1]): 0 for cases with no inflammatory cells; 1 for cases with observable macrophages, lymphocytes, and plasma cells; 2 for cases with macrophages, plasma cells, eosinophils, and neutrophils; and 3 for cases with inflammatory cell infiltration and microabscess formation. All scores were evaluated by two pathologists from our university. Five fields under high-magnification microscopy were randomly selected from each pathological section for scoring, and the average score of each rat was used as the final score.

### 2.5. Sirius Red Picric Acid Staining

More than 5 pathological sections were randomly selected for Sirius red picric acid staining using the experimental method. Staining was performed with 0.1% Sirius red picric acid (Direct Red 80; Sigma-Aldrich, St. Louis, MO, USA), followed by counterstaining with hematoxylin. Five high-magnification fields were randomly selected from each pathological section to measure the width of collagen tissue by using Image-Pro Plus 5.0 software (Leica Qwin. Plus, Leica Microsystem Imaging Solutions Ltd., Cambridge, UK). The average thickness of the adhesive area of each tissue was considered the thickness of the adhesive area of each tissue.

### 2.6. Immunohistochemistry

In this study, immunohistochemistry was performed with a SABC kit (Maxim, Fuzhou, China). All experimental procedures were performed following the operation manual's instructions. The sections were incubated with an *α*-SMA primary antibody (GB13044, Servicebio, Hubei, China, 1 : 200), MMP9 primary antibody (GB13044, Servicebio, Hubei, China, 1 : 200), and Nrf-2 primary antibody (GB13044, Servicebio, Hubei, China, 1 : 200) at 4°C overnight. Subsequently, the sections were incubated with a ubiquitinated secondary antibody at room temperature for 30 min, followed by streptavidin peroxidase for 30 min at the same temperature. Then, DAB development, hematoxylin counterstaining, further dehydration, and mounting were performed. Five high-magnification fields were randomly selected from each section for immunohistochemistry scoring to obtain the average of all the sections of each tissue as the final score. The sections were scored as follows: 0 represented tissues with no expression, 1 represented tissues with weakly positive expression, 2 represented tissues with positive expression, 3 represented tissues with strongly positive expression, and 4 represented tissues with extremely abundant expression.

### 2.7. Western Blot

Protein extraction from tissue samples was performed using the RIPA Protein Extraction Kit (Thermo Fisher Scientific, USA) following the operation manual. Western blotting was performed as described in the literature [11]. The extracted proteins were subjected to 12% sodium dodecyl sulfate polyacrylamide gel electrophoresis and then transferred to a PVDF membrane. After blocking with 5% milk for 1 h, the membrane was incubated with the primary antibody at 4°C overnight. The primary antibodies included anti-NF-*κ*B (catalog no. Ab16502, Abcam, Cambridge, UK, 1 : 1000 dilution), anti-NF-*κ*B (phospho S536) (catalog number ab86299, Abcam, Cambridge, 1 : 1000 dilution), and anti-b-GAPDH (sc-47778, Santa Cruz Biotechnology, 1 : 1000 dilution). After incubation, the PVDF membrane was rinsed and then incubated with the secondary antibody for 1 h at room temperature. Then, the protein expression was detected by using a chemiluminescence detection system (Millipore, Billerica, MA, USA).

### 2.8. ELISA

Enzyme-linked immunosorbent assay (ELISA) was performed according to the manufacturer's description. The indexes included tissue-type plasminogen activator (t-PA) (CSB-E07917r, Shanghai Ximei Chemical Co. Ltd., Shanghai, China), plasminogen-activating inhibitor (PAI-1) (CSB-E07948r, Shanghai Ximei Chemical Co. Ltd., Shanghai, China), TNF-*α* (88-7340, Thermo Fisher Scientific, Waltham, America), and TGF-*β*1 (CSB-E04727r, Shanghai Ximei Chemical Co. Ltd., Shanghai, China).

### 2.9. Isolation and Culture of RPMCs and of ROS

The isolated and cultured rat primary peritoneal mesothelial cells (RPMCs) were performed as previously described [14]. Male SD rats weighing 150-250 g were intraperitoneally injected with 25 mL of 0.25% trypsinase-0.02% EDTA-Na2. The fluid in the abdominal cavity was collected after 30 min and centrifuged at 1000 rpm for 10 min. The cells were cultured in 15% (*v*/*v*) FBS DMEM/F12 medium in 25 cm^2^ tissue culture flasks at 37°C in a humidified 5% CO_2_ atmosphere. The cells were passaged every 3-5 days, and RPMCs from the second and third passages at 80% confluence were used for the following experiments. Before quantifying the ROS levels, the RPMCs (5 × 10^6^) were grown on cover slips. Then, the cells in the DHI-treated group were kept in 2% DHI solutions for 24 hours, while the control group was treated with the same dose of PBS. Then, both groups were treated with 50 *μ*M/L H_2_O_2_ (Sigma Chemical Co., St. Louis, MO, USA) for 1 hour. ROS detection experiments were performed as previously described [15] by a ROS assay kit (KGT010-1, Nanjing KeyGen Biotech. Co. Ltd, China). After washing three times, the cover slips were incubated with 10 *μ*M/L DCFH-DA serum-free DMEM/F12 medium for 30 min in a 37°C environment in the dark. Then, the cells were washed again and mounted on a microslide. The intracellular ROS levels were then detected by a confocal ultraspectral Leica microscope at an excitation wavelength of 488 nm and an emission wavelength of 525 nm.

### 2.10. The Detection of ROS in Adhesion Tissues

The tissues stored at -80°C were prepared into consecutive frozen sections. At least four sections were used to detect the ROS in each tissue. After the sections were slightly dehydrated, the ROS dyeing solution (catG0002, Wuhan Servicebio Technology Co. Ltd., China) was added to the sectioned tissue, and the sections were incubated in a 37°C environment in the dark for 30 min. After washing three times, the slices were incubated in DAPI dyeing solution (catG1012, Wuhan Servicebio Technology Co. Ltd., China) for 10 min at room temperature. Then, the slices were washed three additional times and dried with resistance to fluorescence quenching after sealing seal tablets. These slices were observed by fluorescence microscopy, and images were collected. The ROS expression was calculated as the ratio of ROS-positive nuclei to total nuclei.

### 2.11. Statistical Methods

All data in this study were analyzed using SPSS 18.0 (Chicago, IL, USA), and all data are expressed as the mean ± standard deviation. The normally distributed data were compared with a *t* test or one-way ANOVA and LSD, and the data without a normal distribution were analyzed using the Kruskal-Wallis test. The counting data were processed using Fisher's exact test. A *P* value less than 0.05 was considered statistically significant.

## 3. Results

### 3.1. DHI Treatment Alleviates Intra-abdominal Adhesion

**Figure 1:**
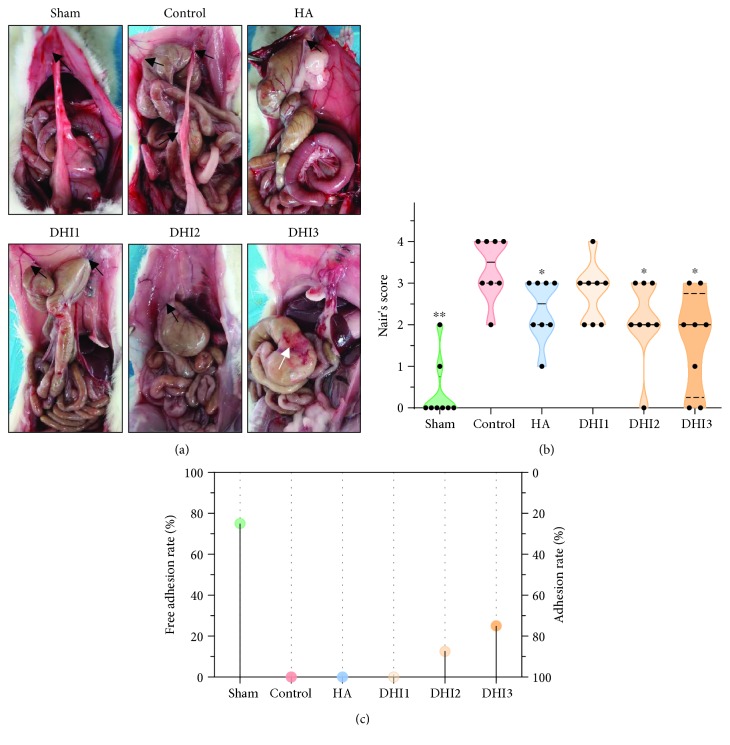
The adhesion formation conditions of different groups (*n* = 8). (a) In the sham-operated group, intra-abdominal adhesions occurred in two rats only. All rats in the control group exhibited severe adhesion. All rats in the HA-treated and DHI1 groups had moderate and loose adhesion bands. The adhesions of the rats in the DHI2 and DHI3 groups were lighter than those in the control group. One rat in the DHI2 group and two rats in the DHI3 group had no adhesion formation. The black arrows indicate adhesion on the abdominal wall. The white arrows indicate the injured serosal surface of the cecum without adhesion formation. (b) The Nair score for each group (compared with the control group, ^∗^*P* < 0.05 and ^∗∗^*P* < 0.01, abnormal distribution, Kruskal-Wallis test). (c) The nonadhesion rate of each group.

No rat died or had severe postoperative complications during the experiment. Seven days after the operation, intra-abdominal adhesion was assessed by Nair's scoring system, and the results are presented in Figure 1. The rats in the control group had extensive adhesion, and the adhesion bands were thick. Compared to the control group, both the HA- and DHI-treated groups had a moderate degree of adhesion, and the adhesion bands appeared smaller and looser in the HA- and DHI-treated groups than the control group (Figure 1(a)). Although the adhesion score exhibited a decreasing tendency in the HA- and DHI-treated groups compared with the control group, only the DHI3 group showed a statistically significant difference (Figure 1(b); *P* < 0.05). However, the preventive effect of DHI was not significantly different from the HA group. Only two rats had loose incision adhesion in the sham group, while only one rat in the DHI2 group and two rats in the DHI3 group had no adhesion. All rats in the other groups had adhesion (Figure 1(c)).

### 3.2. DHI Inhibits the Inflammatory Response in Adhesion Tissue

**Figure 2:**
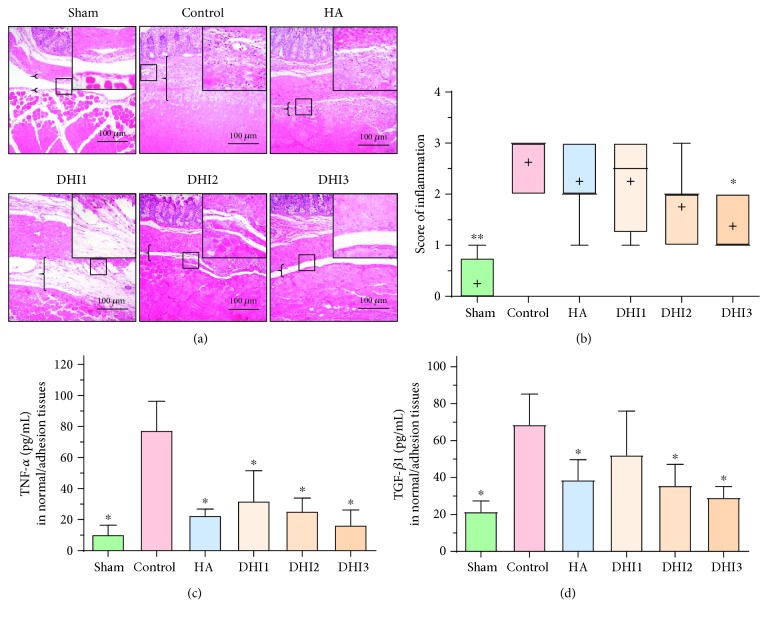
DHI treatment can reduce inflammation in adhesion tissue in the rat model 7 days after operation (*n* = 8; compared with the control group, ^∗^*P* < 0.05 and ^∗∗^*P* < 0.01). (a) HE staining of each group at 100x magnification. The top right corner is shown at 200x magnification. The brackets indicate tissue with adhesion. (b) The inflammatory score of each group based on HE staining (compared with the control group, ^∗^*P* < 0.05 and ^∗∗^*P* < 0.01, normal distribution, one-way ANOVA). (c) The TNF-*α* expression in each group (compared with the control group, ^∗^*P* < 0.05, abnormal distribution, Kruskal-Wallis test). (d) The TGF-*β* expression in each group (compared with the control group, ^∗^*P* < 0.05, abnormal distribution, Kruskal-Wallis test).

To determine the possible mechanism by which DHI reduces postoperative abdominal adhesion, we detected inflammation-related indicators. HE staining showed that the inflammation score of the DHI treatment groups that administered the highest doses of DHI was decreased compared with that of the control group (Figures 2(a) and 2(b)). ELISA detection of TNF-*α* and TGF-*β*1 levels in the adhesion tissue showed that TNF-*α* was significantly decreased in the DHI- and HA-treated groups compared with the control group (Figure 2(c); *P* < 0.05), while TGF-*β*1 was decreased in the HA- and middle- and high-DHI-treated groups compared with the control group (Figure 2(d); *P* < 0.05).

### 3.3. DHI Could Decrease Collagen Deposition and Promote Fibrinogenolysis in the Adhesion Tissue

**Figure 3:**
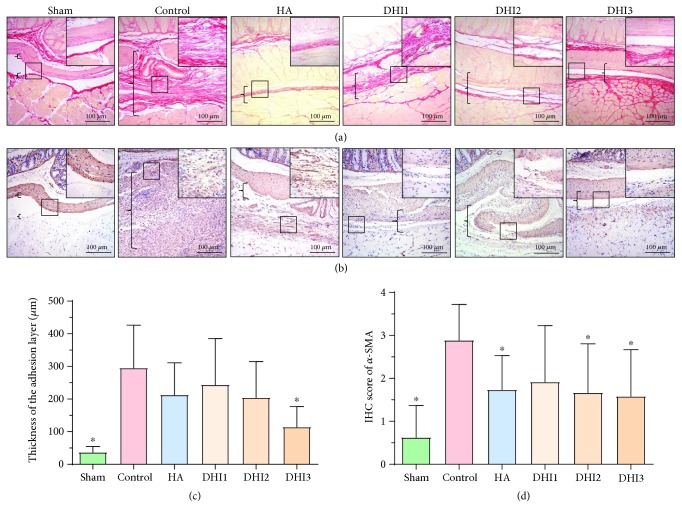
DHI treatment attenuated collagen deposition in the adhesion tissue of the rat model 7 days after operation (*n* = 8; compared with the control group, ^∗^*P* < 0.05 and ^∗∗^*P* < 0.01). (a) Sirius red picric acid staining of each group at 100x magnification. The top right corner is shown at 200x magnification. The brackets indicate tissue in the adhesion area. (b) *α*-SMA staining of each group at 100x magnification. The top right corner is shown at 200x magnification. The brackets indicate tissue in the adhesion area. (c) Adhesion thickness in each group as assessed by Sirius red picric acid staining (compared with the control group, ^∗^*P* < 0.05, abnormal distribution, Kruskal-Wallis test). (d) *α*-SMA staining scores of each group (compared with the control group, ^∗^*P* < 0.05, normal distribution, one-way ANOVA).

**Figure 4:**
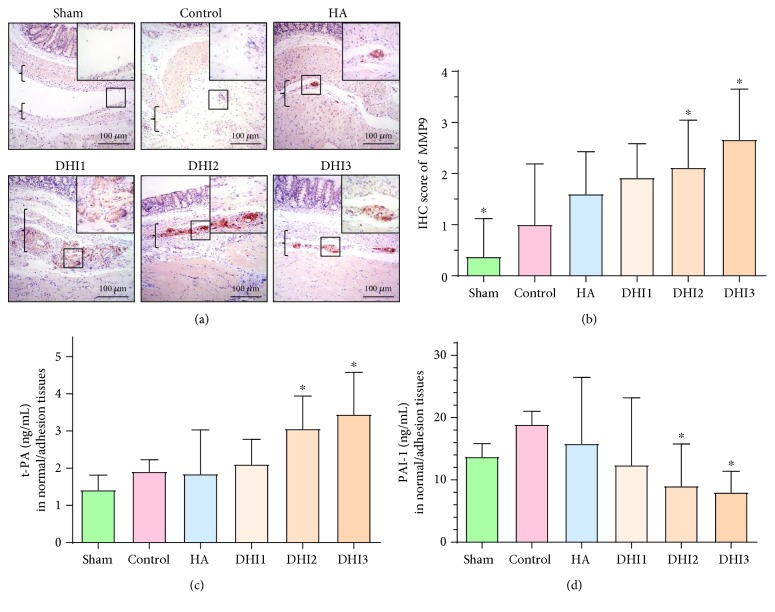
DHI treatment promoted fibrinogenolysis in the adhesion tissue of the rat model 7 days after operation (*n* = 8; compared with the control group, ^∗^*P* < 0.05). (a) MMP9 staining of each group at 100x magnification; the top right corner is shown at 200x magnification. The brackets indicate tissue in the adhesion area. (b) MMP9 staining scores of each group (compared with the control group, ^∗^*P* < 0.05, normal distribution, one-way ANOVA). (c) The t-PA expression in the adhesion tissue of each group (compared with the control group, ^∗^*P* < 0.05, abnormal distribution, Kruskal-Wallis test). (d) The PAI-1 expression in the adhesion tissue of each group (compared with the control group, ^∗^*P* < 0.05, abnormal distribution, Kruskal-Wallis test).

Collagen deposition is one of the most important reasons for adhesion formation. Sirius red staining, which reflects the collagen density of adhesion tissues, demonstrated that the fibrin thickness of the adhesion tissue in the DHI3 group was decreased compared with the control group (Figures 3(a) and 3(c); *P* < 0.05). Immunohistochemical staining of *α*-SMA (marker of myofibroblasts) showed that the *α*-SMA levels in the HA-treated group, DHI2 group, and DHI3 group were reduced compared with the control group (Figures 3(b) and 3(d); *P* < 0.05). To investigate the mechanism by which DHI reduced collagen deposition, we quantified the matrix metalloproteinase system indicator MMP9 and the fibrinogenolysis system indexes t-PA and PAI-1. The results demonstrated that the expression of MMP9 and t-PA in the adhesion tissues was increased in the DHI2 and DHI3 groups compared with the control group (Figures 4(a)–4(c); *P* < 0.05), whereas the level of PAI-1 was decreased in the DHI2 and DHI3 groups compared with the control group (Figure 4(d); *P* < 0.05).

### 3.4. DHI Reduces Oxidative Stress in Adhesion Tissues

**Figure 5:**
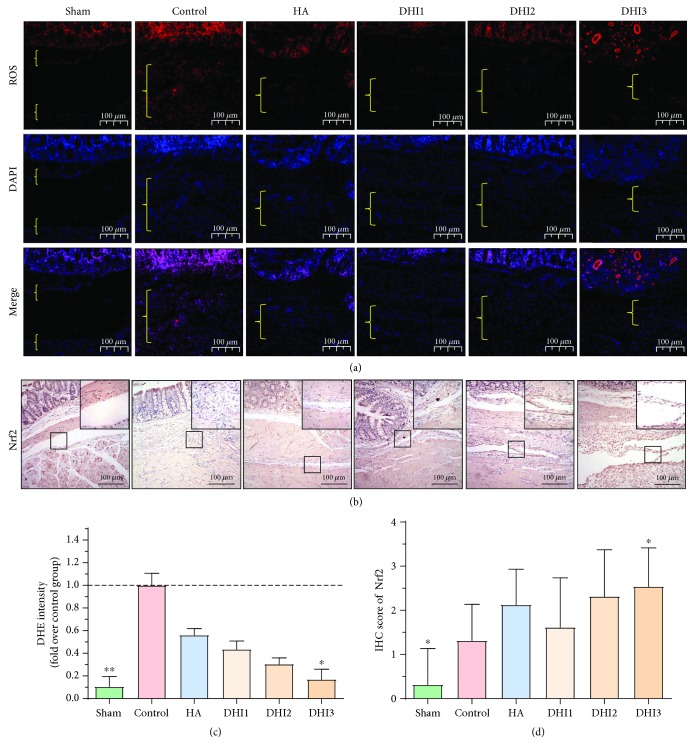
DHI treatment reduces oxidative stress in the adhesion tissues of the rat model 7 days after operation (*n* = 8; compared with the control group, ^∗^*P* < 0.05). (a) Representative ROS level of each group at 100x magnification, and the area between the bracket indicates the tissue in the adhesion area. (b) Nrf2 staining scores of each group at 100x magnification. The top right corner is shown at 200x magnification. (c) Relative ROS expression in the adhesion tissue of each group (compared with the control group, ^∗^*P* < 0.05, abnormal distribution, Kruskal-Wallis test). (d) Nrf2 staining scores of each group (compared with the control group, ^∗^*P* < 0.05, normal distribution, one-way ANOVA).

ROS levels play an important role in adhesion formation [16]; thus, we measured a ROS-related index in vivo. The ROS level in the adhesion tissues was remarkably reduced in the DHI3 group (Figures 5(a) and 5(c); *P* < 0.05). Furthermore, we detected the expression of the antioxidative stress index Nrf2 in the adhesion tissues and found that the Nrf2 level was also increased in the DHI3 group compared with the control group (Figures 5(b) and 5(d); *P* < 0.05).

### 3.5. DHI Reduces Oxidative Stress in RPMCs

**Figure 6:**
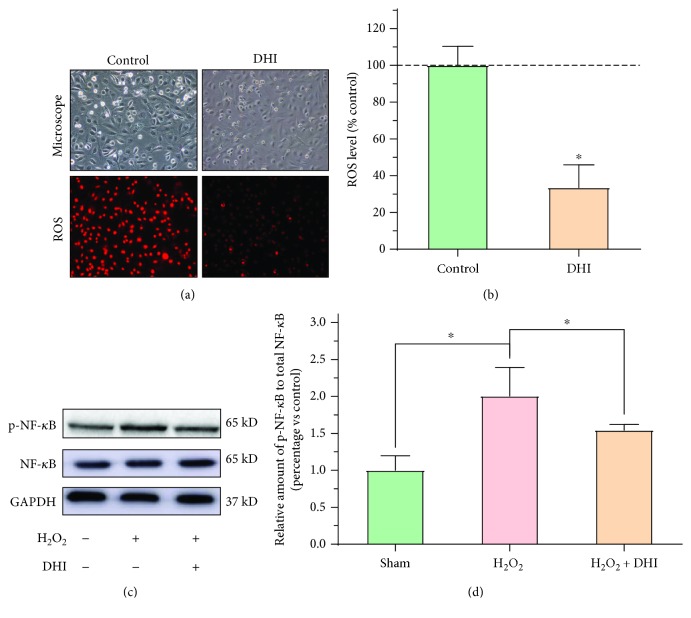
DHI treatment reduces oxidative stress in RPMCs (compared with the control group, ^∗^*P* < 0.05 and ^∗∗^*P* < 0.01). (a) Representative cellular morphology and ROS expression in DHI-treated and control cells at 100x magnification. (b) The ROS level in DHI-treated and control cells (compared with the control group, ^∗^*P* < 0.05, normal distribution, *t* test). (c) Western blot detection of NF-*κ*B and phosphorylated NF-*κ*B expression in the different DHI- and H_2_O_2_-treated groups. (d) The ratio of the relative expression of phosphorylated NF-*κ*B to total NF-*κ*B in each group (compared with the control group, ^∗^*P* < 0.05, ^∗∗^*P* < 0.01, normal distribution, one-way ANOVA).

To further verify that DHI reduces ROS in adhesion tissue, we detected the ROS expression in RPMCs, as shown in Figures 6(a) and 6(b). When the samples were treated with the same dose of H_2_O_2_, the 2% (*v*/*v*) DHI-treated group had a lower ROS level than the control group (Figures 6(a) and 6(b); *P* < 0.05). To further illustrate the mechanism by which DHI decreased ROS levels, we detected the NF-*κ*B expression in each group. The level of phosphorylated NF-*κ*B was significantly decreased in the DHI-treated groups compared with the control group (Figures 6(c) and 6(d); *P* < 0.05).

## 4. Discussion

The traditional Chinese medicine DHI is extracted from *S. miltiorrhizae* Radix and *C. tinctorii* Flos, and various studies have demonstrated that DHI can be used to treat cerebral ischemia and heart diseases [17], as well as ischemia-reperfusion injury [18]. Herein, we demonstrated that DHI can be used as a potential drug to prevent postoperative adhesion by reducing inflammatory reactions, decreasing fibrosis, and alleviating oxidative stress.

The formation of intestinal adhesion is complex. Initially, injury causes and amplifies inflammation, followed by collagen deposition and adhesion [19]. In this process, the repair of the local peritoneal tissue repair also plays an important role; if the injured area can be repaired in a timely manner, limited adhesion develops. Thus, preventing the formation of abdominal adhesion after surgery requires all of these factors to be considered, and simple physical barriers may not be satisfactory. This problem has been verified in many clinical studies [20].

It is widely acknowledged that postoperative adhesion formation is stimulated by inflammation and oxidative damage [5, 21]. Trauma and injury after operation will inevitably lead to oxidative stress reactions and inflammation, and the increase in ROS can accelerate the amplification of inflammation and damage mesothelial cells [22]. To determine whether the anti-inflammatory activity of DHI exerts a protective role in adhesion formation in a rat model, this prospective study was designed. HE staining was used as an index for inflammation. The results demonstrated that DHI treatment reduced inflammation. Additionally, the levels of cytokines such as TGF-*β*1 and TNF-*α* in adhesion tissues were measured, and the results demonstrated that the DHI-treated groups had lower levels of TGF-*β*1 and TNF-*α* expression in adhesion tissues than the control group. These results suggest that DHI alleviates the formation of abdominal adhesion by reducing inflammation in the adhesion tissue.

The interaction between oxidative stress and inflammation plays a critical role in adhesion formation; high concentrations of ROS can injure peritoneal mesothelial cells and promote inflammation. A previous study demonstrated that DHI reduces oxidative stress by activating superoxide dismutase (SOD) and reducing the production of malondialdehyde (MDA) in both myocardial ischemia/reperfusion (MI/R) and acute lung injury (ALI) mouse models [23]. Here, we demonstrated that DHI can reduce the level of ROS in both adhesion tissue and H_2_O_2_-treated mesothelial cells. To further illustrate this effect in adhesion tissue, we quantified the expression of Nrf2, which is a transcription factor that regulates an expansive set of antioxidant-related genes and acts in synergy to remove ROS through sequential enzymatic reactions [24]. We found that the expression of Nrf2 in the adhesion tissue of DHI-treated rats was increased compared with that of control rats. To explore its mechanism, we detected the expression of the important inflammation factor NF-*κ*B in H_2_O_2_- and DHI-treated RPMCs. The results showed that DHI treatment can decrease the NF-*κ*B expression in mesothelial cells. This effect was consistent with previous studies [10, 25]. Therefore, these results suggest that one possible mechanism for the DHI-mediated reduction of inflammation in adhesion tissue is the inhibition of ROS via a decrease in NF-*κ*B phosphorylation.

Another explanation for the DHI-mediated prevention of adhesion formation may be the reduction of collagen deposition and promotion of fibrinolysis in adhesion tissues. Within seven days after operation, the balance between fibrin deposition and degradation determines whether normal peritoneal healing or adhesion formation occurs. After the operation, inflammation increased, which resulted in fibrinous exudate and fibrin formation. Fibrin forms as a result of the activation of a coagulation cascade. However, owing to the activation of the fibrinolytic and extracellular matrix system (MMPs), any intra-abdominal fibrin deposits must be lysed. t-PA is a major plasminogen activator and has a high affinity for fibrin, while MMP9 is an important enzyme classified as an MMP. Plasminogen activation is impaired by PAI-1 and its analog through the formation of inactive complexes [26]. Many studies have demonstrated that DHI can reduce collagen deposition [27]. In our study, we found that DHI increased the t-PA expression and decreased the PAI-1 expression in adhesion tissue. In addition, DHI treatment promoted the MMP9 expression, which resulted the enhancement of the fibrinolytic and MMP systems.

The strategies for the prevention and treatment of postoperative adhesion are various [3]. While base on the mechanism for adhesion development, these methods can be mainly classified as the following. The first one is using the physical barrier such as membrane or gel; these barriers can separate the injured peritoneal tissue which can reduce the adhesion formation [19, 28]. The second way for postoperative adherence prevention is alleviating the inflammation and its enlargement; controlling the inflammation reaction can reduce the vascular permeability and tissue damage [29]. Thirdly, reducing the fiber deposition or promoting adhesion tissue fiber dissolving is very important, especially during 3 to 5 days after the operation [30]. The fourth one is protection of peritoneal mesothelial cells. After the operation, the mesothelial cells will not only be injured or dead, it can also be transferred into fibroblasts thus promote the adhesion formation; moreover, the lack of peritoneal mesenchymal cell and exposure of subperitoneal collagen tissue can promote the formation of adhesion [31, 32]. Lastly and the most important methods to prevent adhesion is reduction of surgical damage, exact hemostasis, and removal of blood clots during the operation; besides, surgeons should avoid residual foreign substance [33].

In this study, we demonstrated that DHI can potentially be used as a drug to prevent the formation of postoperative adhesion. However, there are several limitations in our study. First, this study is based on an animal model, and the effect of DHI on human adhesion formation requires further investigation. The other limitation is that because of the complexity of DHI, the pharmacological action and toxicity of DHI are not fully understood; thus, additional studies investigating the individual components of DHI on adhesion prevention are required.

## 5. Conclusion

DHI can inhibit the formation of intra-abdominal adhesion in a rat model by inhibiting inflammation, collagen deposition, and oxidative stress. DHI may serve as a promising drug for preventing intra-abdominal adhesion.

## Data Availability

The data used to support the findings of this study are included within the article.

## Supplementary Materials

Supplementary Materials Supplemental Table 1: Nair et al. scoring system. Supplemental Table 2: histopathological criteria for inflammatory score.
