# Supplementary material for: Danhong Injection Alleviates Postoperative Intra-abdominal Adhesion in a Rat Model
Source: Oxid Med Cell Longev. 2019 Aug 19;2019:4591384. doi: 10.1155/2019/4591384 (PMC6721271; doi:10.1155/2019/4591384)
Supplement: Supplementary Materials — Supplemental Table 1: Nair et al. scoring system. Supplemental Table 2: histopathological criteria for inflammatory score. [file 4591384.f1.pdf]

Supplemental Table 1. Nair et al. scoring system

0	No adhesions
1	Between viscera or between visceral viscus and abdominal wall (one band)
2	Between viscera or between visceral viscus and abdominal wall (two bands)
3	Between viscera, between visceral viscus and abdominal wall (more than two bands) or multiple intestinal adhesions without adhesion to the abdominal wall
4	Direct adhesion of the viscera to the abdominal wall (number and size of bands not important)

Supplemental Table 2. Histopathological criteria for inflammatory score

Score	Degree of inflammation
0	No inflammation
1	Giant cells, lymphocytes, and plasma cells
2	Giant cells, plasma cells, eosinophils, and neutrophils
3	Inflammatory cell infiltration and microabscess formation
